# Characteristic analysis and surgical exploration for acetabular roof fractures: Multicenter retrospective cohort study

**DOI:** 10.1371/journal.pone.0317932

**Published:** 2025-02-06

**Authors:** Ruipeng Zhang, Yingchao Yin, Wei Chen, Yan Zhuang, Shicai Fan, Chengla Yi, Gang Lyu, Longpo Zheng, Xiaodong Guo, Ming Li, Guangyao Liu, Zhiyong Hou, Yingze Zhang

**Affiliations:** 1 Department of Orthopaedics, Hebei Medical University Third Hospital, Shijiazhuang, China; 2 Department of Orthopedic Trauma, Honghui Hospital, Xi’an Jiaotong University, China; 3 Department of Orthopaedic, The Third Affiliated Hospital of Southern Medical University, China; 4 Department of trauma Surgery, Tongji Hospital, Tongji Medical College, Huazhong University of Science and Technology, China; 5 Department of Orthopaedic, Affiliated Hospital of Traditional Chinese Medicine of Xinjiang Medical University, China; 6 Department of Orthopedics, Shanghai Tenth People’s Hospital, School of Medicine, Tongji University, China; 7 Department of Orthopaedics, Union Hospital, Tongji Medical College, Huazhong University of Science and Technology, China; 8 Department of Traumatic Orthopaedics, The Medical College of Ningbo University Affiliated Ningbo No.6 Hospital, China; 9 Department of Orthopedics, China−Japan Union Hospital of Jilin University, China; Menoufia University, EGYPT

## Abstract

**Background:**

Acetabular roof was a crucial structure for maintaining the stability of hip joint; however, its important role was not especially emphasized in the Letournel-Judet classification system. Acetabular roof was segmented into the roof column and roof wall in Three-column classification and fracture in this area alone was defined as A3 injury. The purpose of this study was to explore the characteristics and surgical strategy of A3 injury.

**Methods:**

Patients with roof column/wall fractures received surgical management from January 2015 to 2019 January at nine level-1 trauma centers were retrospectively analyzed. Fracture data, surgical incision, operation time, blood loss, fracture healing and relevant complications were recorded to explore fracture characteristics and appropriate surgical strategy. Reduction quality was assessed based on postoperative radiographic examination. Merle d’Aubigné score was used to assess the functional outcome during the follow-up.

**Results:**

A total of 60 patients met the inclusion criteria in this study. Mean operation time was 112.83±21.77 min, and mean intraoperative blood loss was 396.67±182.00 ml. Satisfactory reduction quality was obtained in 49 cases (81.67%). All fractures healed well at an average mean of 3.07 months. Satisfactory outcomes were obtained in 46 cases (76.67%), and mean Merle d’Aubigné score was 15.53±1.33 points at the final follow-up. Reduction quality and functional outcome showed no statistical difference in three subtypes (*P*<0.05). Reduction quality and functional outcome presented positive correlation in three subtype fractures (*P*<0.05). The complication rate was 11.67% (7/60) in this study.

**Conclusion:**

The injury mechanism of A3 injury was the direct impaction from femoral head on acetabular roof. Reduction and fixation of A3 injury were technique demanding, and poor prognosis may be accompanied even treated by experienced surgeons. Appropriate surgical strategies (Table 5) based on fracture characteristics in three subtypes of A3 injury were the premise of accepted prognosis.

## Introduction

Acetabular fractures were divided into five elementary and five associated fracture types in the Letournel-Judet classification, which was employed by most orthopedic surgeons to diagnose acetabular fractures [1–3]. Acetabular roof played an extremely important role in the maintenance of hip stability, and surgical management was preferred when its integrality was destroyed [4]. However, its vital role was not emphasized in Letournel-Judet classification system. What’s more, classification of roof acetabular fracture was controversial [3, 5]. Lenarz CJ defined this fracture type as an anterior column fracture without the involvement of the pelvic brim [5]. Nevertheless, the same fracture type was classified as unusual variant of posterior wall fracture with extension into iliac wing [6]. The controversial classification may have a negative influence on its diagnosis and communication among orthopedic surgeons. Moreover, surgical strategy may be distinct because of its controversial classification. For example, Kocher-Langenbeck (KL) approach was recommended for posterior wall fracture (described by Letournel E); however, an anterior incision was preferred for anterior column fractures (defined by Lenarz CJ) [4, 6–8].

The crucial role of acetabular roof was emphasized in Three-column classification system [4]. It was divided into the roof wall and roof column in this novel classification. Based on the extent of fracture line involvement, roof injury was classified as roof wall fracture (A3.1), roof column fracture (A3.2), and complicated roof column fracture (A3.3), resolving the controversial classification problem in the traditional classification system [4]. However, fragment characteristics and treatment in roof area were not emphasized in that study. This study retrospectively analyzed the clinical outcome of patients with acetabular roof fractures to explore the fracture characteristics and their appropriate surgical strategies

## Materials and methods

### Patients

Patients with roof column/wall acetabular fractures received surgical management from January 2015 to 2019 January at nine Level-1 trauma centers were retrospectively analyzed. The inclusion criteria were as follows: 18–80 years, acute (<14 days) closed roof column/wall acetabular fractures (A3.1, A3.2 or A3.3) in Three-column classification, normal activity of the affected hip before the injury, received surgical reduction and internal fixation, and completed at least 24 months of follow-up. The exclusion criteria were as follows: open fractures, pathologic fractures, limited range of motion (ROM) of the hip joint before the injury, conservative treatment, risk factors affecting bone healing (such as smoking and metabolic diseases) and noncompletion of the two-year follow-up. Relevant data of this study were assessed on March 1, 2022. Authors had no information that could identify individual participants during or after data collection. This study was carried out in accordance with The Code of Ethics of the World Medical Association [9]. This retrospective study was performed in compliance with relevant laws and institutional guidelines and have been approved by the appropriate institutional committee of Third Hospital of Hebei Medical University (2017-005-1). As a retrospective study, all data were fully anonymized before we accessed them and informed consent was waived by ethics committee after profound discussion.

### Surgical techniques

All surgical procedures were performed by experienced surgeons (each with over 20 years of experience in the treatment of acetabular fractures) from Level-1 trauma centers. Different surgical approaches and fixation methods were selected based on the fracture types in Three-column classification [4].

KL technique used to be applied to manage the roof wall fragments. A flexed knee was maintained throughout the KL procedures to reduce the iatrogenic injury rate of the sciatic nerve. The external rotators (including piriformis, superior and inferior gemelli, and obturator internus) were partially cut from the greater femoral trochanter to obtain a better surgical vision, which was very helpful to manage displaced fragments. Compared with typical posterior wall detachment, the level of displaced fragments in roof column/wall fractures was cranial. Then, auxiliary trochanteric osteotomy may be employed to better expose the fracture line in the acetabular roof. Plating and/with lag screw should be performed after a satisfactory reduction quality was obtained.

Iliac fossa approach (lateral window of the ilioinguinal approach) could be applied to treat roof column fragments. The cephalad displaced roof column fragment was reduced with the help of assistant traction, and temporary fixation was accomplished by Kirschner wires. Plating and screw insertion were performed to achieve the fixation of the fragments. For patients with a lower fracture line, the ilioinguinal technique was an alternative choice.

Iliofemoral approach had a relatively large exposure area and can fully exposed all the displaced fragments of acetabular roof fractures. Then, all subtype fractures could be managed by iliofemoral technique. It was an option when other relative less invasive approaches (KL or Iliac fossa) failed to reduce and fix the displaced fragments. Lateral femoral cutaneous nerve (LFCN) should be protected and retracted medially during surgical procedure. Treatment of complicated roof column fractures (A3.3) could be accomplished through combined (iliac fossa and KL) or iliofemoral techniques. Management of roof column fragments should be superior to roof wall detachment for patients with A3.3 injury. For patients with articular compression, detached surface should be reduced regarding femoral head as a landmark. Bone graft under articular surface and buttress fixation should be performed for patients with bone defects.

### Outcome measures and statistical analysis

Surgical approach, operation time, blood loss and relevant complications were recorded. Reduction quality of displaced fragment was assessed by postoperative radiographs according to the criteria described by Matta, which could be classified as excellent (0-1mm displacement), imperfect (2-3mm displacement), and poor (>3mm displacement) [10]. Excellent and imperfect were regarded as satisfactory reduction quality in this study. Fracture healing and functional recovery were evaluated at each follow-up, which were conducted at one, three, and six months postoperatively and every six months thereafter. Complications (including heterotopic bone, major neurovascular injuries, infection, etc) were recorded in this study. Merle D’Aubigné score was employed to assess the functional outcome at the final follow-up, which could be classified as excellent (18), good (15–17), fair (13–14), or poor (<13) [11]. Excellent and good were regarded as satisfactory outcome in this study. Relevant data were processed with SPSS version 23.0 software (SPSS, Chicago, Illinois).

## Results

A total of 2569 patients with acetabular fractures treated in the attending institutions during the study period. There were 102 cases (3.97%) with acetabular roof column/wall fractures. A total of 42 patients were excluded in this study, including 12 cases for noncompletion of the two-year follow-up, 9 cases for conservative treatment, 5 patients for limited ROM, and 16 cases for risk factors affecting bone healing. A total of 50 male and 10 female patients were included in this study, including 13 cases with roof wall fracture (A3.1), 24 with roof column fractures (A3.2), and 23 complicated roof column fractures (A3.3) (S1 Data). The mean age of patients actually included in this study was 42.52 years (range, 22–75 years). There were 31 fractures on the right and 29 fractures on the left. Relevant fracture types and surgical procedures performed were presented in Table 1. The average follow-up time of this study was 26.52±2.78 months.

**Table 1:** Patient distribution and relevant surgical approach.

Fracture type (number)	Surgical procedure	Patient number
A3.1 (13)	KL (Fig 1)	3
	Iliofemoral (Fig 2)	10
A3.2 (24)	Ilioinguinal (Fig 3)	7
	Iliofemoral (Fig 4)	2
	Iliac fossa (Fig 5)	15
A3.3 (23)	Combined (Iliac fossa and KL) (Fig 6)	14
	Iliofemoral (Fig 7)	9

Patient distribution and surgical approach performed are presented. KL, Kocher-Langenbeck approach.

Mean operation time was 112.83±21.77 min, and mean intraoperative blood loss was 396.67±182.00 ml. Postoperative radiographic examination showed that satisfactory reduction quality was obtained in 49 cases (81.67%), which consisted of 20 excellent and 29 imperfect patients. Poor reduction was obtained in 11 patients (18.33%). There was no statistical difference in reduction quality among the three subtypes (Table 2) (*P* = 0.922). All fractures healed well at an average mean of 3.07 months. Excellent and good outcomes were obtained in 46 cases (76.67%). The mean Merle d’Aubigné score of all cases included was 15.53±1.33 points at the final follow-up. Mean Merle D’Aubigné score in three subtypes were 15.92±1.66, 15.71±1.23, and 15.13±1.17, respectively, (*P* = 0.164). Reduction quality and functional outcome were analyzed and presented positive correlation in three subtype fractures (Table 3).

**Table 2:** Reduction quality of three subtypes.

Fracture Type	Patient Number		
	Excellent	Imperfect	Poor
A3.1	5	6	2
A3.2	9	11	4
A3.3	6	12	5

There was no statistical difference in reduction quality among the three subtypes (*P* = 0.922).

**Table 3:** Correlation between reduction quality and functional outcome.

Fracture type	Pearson correlation	*P*
A3.1	0.641	0.018
A3.2	0.756	<0.001
A3.3	0.867	<0.001

The reduction quality and functional outcome presented positive correlation in three subtype fractures.

**Fig 1:**
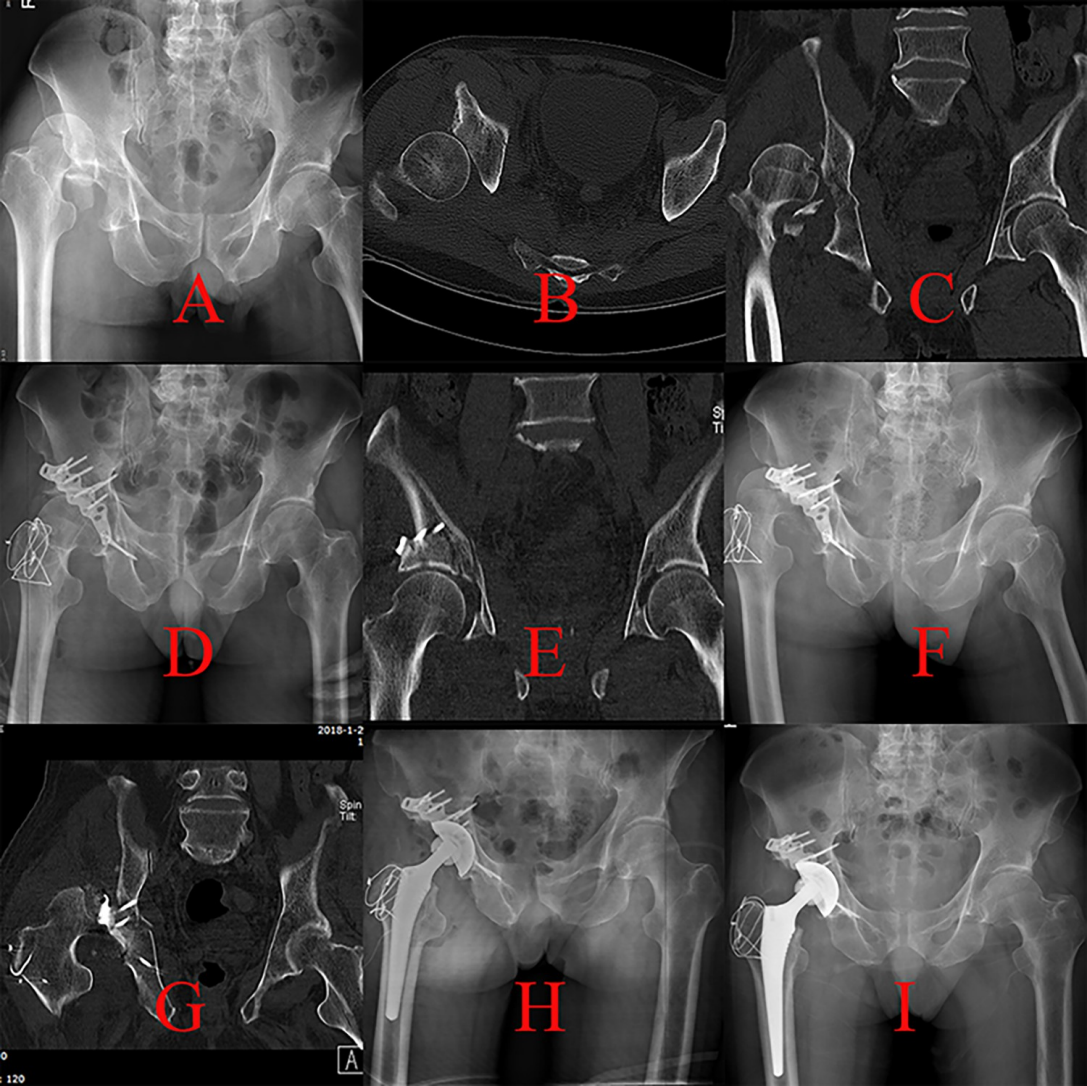
The therapeutic process of a patient with A3.1 injury who underwent the KL procedure was presented. A, cephalad displaced femoral head and displaced roof wall fragment (more cranial than traditional PW fracture) in an anteroposterior (AP) view; B-C, superior posterior dislocation of femoral head on CT scans; D, postoperative AP projection after the KL procedure; E, the femoral head was contained in a concentric acetabulum although there were residual displacements of roof wall fragments on postoperative CT; F, secondary dislocation was noted 3 months postoperatively; G, dislocation and necrosis of affected femoral head on a CT scan; H, AP view after THA surgery; I, AP view 6 months postoperatively.

The complication rate was 11.67% (7/60) in this study (Table 4). Lateral femoral cutaneous nerve (LFCN) injury was observed postoperatively in 4 cases, but the lateral thigh paralysis disappeared after 3 months of conservative treatment. Infection developed in 2 cases, and relevant symptoms disappeared after thorough debridement and antibiotic treatment. Secondary dislocation and femoral head necrosis were noted in a patient with an A3.1 injury after KL technique 3 months postoperatively, then, total hip arthroplasty (THA) was performed (Fig 1). Heterotopic bone and other major neurovascular injuries were not observed in this study. However, these potential complications should be taken seriously.

**Table 4:** Complication of relevant surgical approach.

Complication	Surgical approach	Patient number
LFCN injury	Ilioinguinal	3
	Iliac fossa	1
Infection	Iliofemoral	1
	Combined	1
Femoral head necrosis	KL	1

Complication and relevant surgical approach are presented. LFCN, lateral femoral cutaneous nerve; Combined, iliac fossa and KL approach; KL, Kocher-Langenbeck approach.

## Discussion

Our results showed that surgical treatment of roof column/wall acetabular fracture was technically demanding, and a poor outcome (such as femoral head necrosis) may occur even when the surgical procedures were performed by experienced surgeons (Fig 1). However, accepted prognosis could be gained through appropriate surgical strategies based on fracture characteristics.

Acetabular roof plays an important role in the integrity and stability of the hip joint; however, diagnosis and management strategy of fractures in this region was controversial in the traditional classification system [1, 5, 6, 12]. Then, serious complications, such as instability of the hip joint and traumatic arthritis may be accompanied. The importance of the roof column/wall was emphasized in the Three-column classification [1, 4–6]. Nevertheless, its surgical strategy and postoperative outcome were not further explored. The purpose of this study was to explore their fracture characteristics and the appropriate surgical strategy.

**Fig 2:**
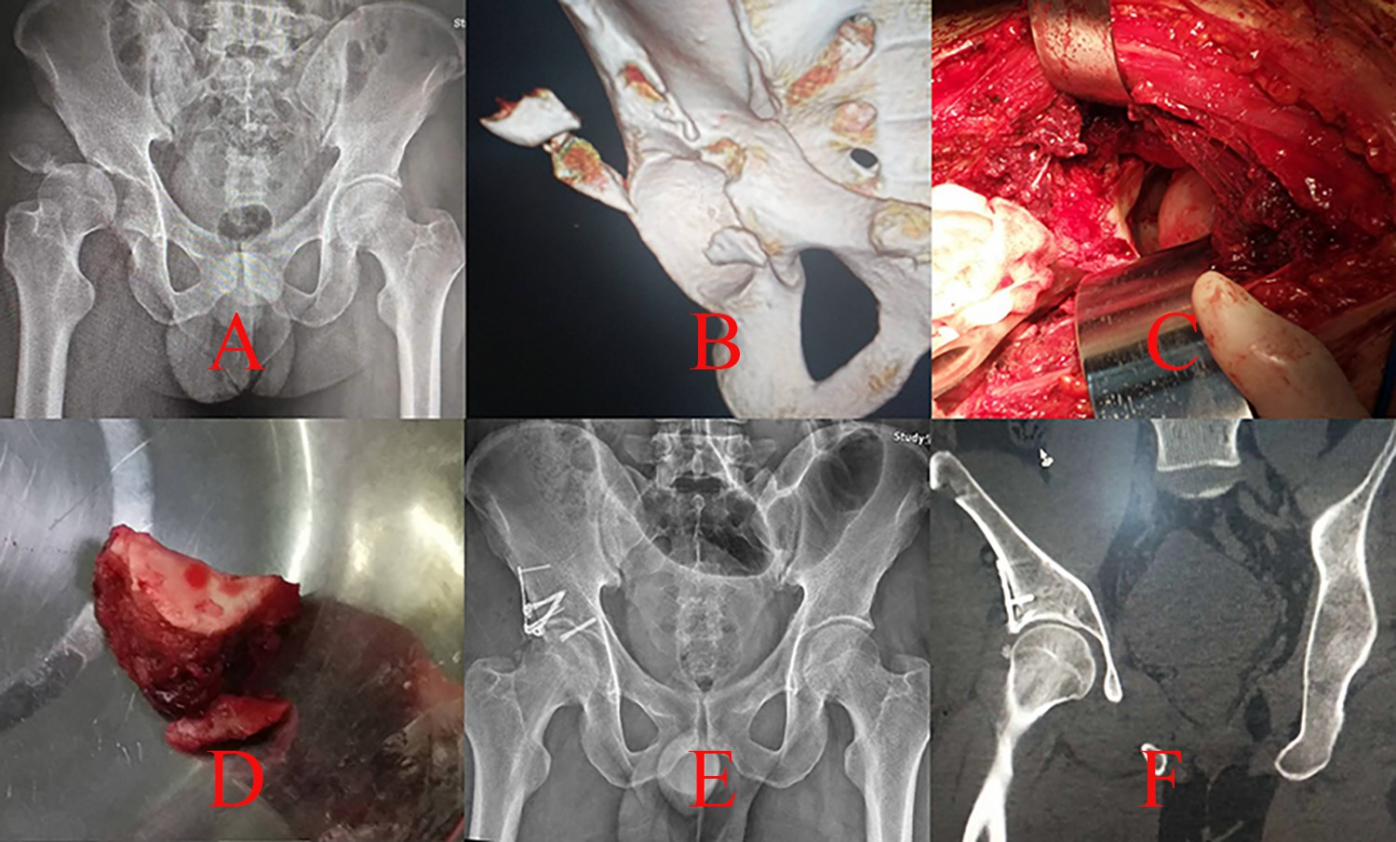
A case with A3.1 injury treated through the iliofemoral technique was presented. A, cephalad displaced femoral head and displaced roof wall fragment in AP view; B, comminuted small roof wall fragments on 3D reconstruction; C, intraoperative view (femoral head and fragments) of iliofemoral procedure; D, displaced roof wall fragment; E, plating with cannulated screws fixation presented and the reduction quality was excellent; F, excellent concentric reduction on postoperative CT scan.

For A3.1 injury, the fracture line only involved the articular surface of the acetabular roof, and a discontinuous iliac crest could not be observed [4]. The injury mechanism of this fracture type was direct impaction from the femoral head on the roof wall. Compared with posterior wall fracture (A2.1 in Three-column classification), the location of the fracture line in A3.1 injury was more cranial because the affected hip joint was more extended during the impaction procedure [13]. Adequate exposure of the relevant fracture lines of A3.1 injury through traditional KL approach was challenging [14]. Auxiliary greater trochanteric osteotomy was performed in three patients by experienced surgeons. One case (33.33%) suffered secondary hip dislocation and femoral head necrosis because of poor reduction and fixation effects. Subsequent THA brought a serious burden on the patient’s body and finances (Fig 1). Each detached fragment during an A3.1 injury involving the articular surface should be properly managed because of its vital role in maintaining hip stability. Thus, the KL technique was not routinely recommended in consideration of its limited exposure for roof wall detachment. Complete exposure of fragments in A3.1 detachment could be achieved through iliofemoral approach, which was another common technique to manage roof wall fracture (Fig 2) [15].

**Fig 3:**
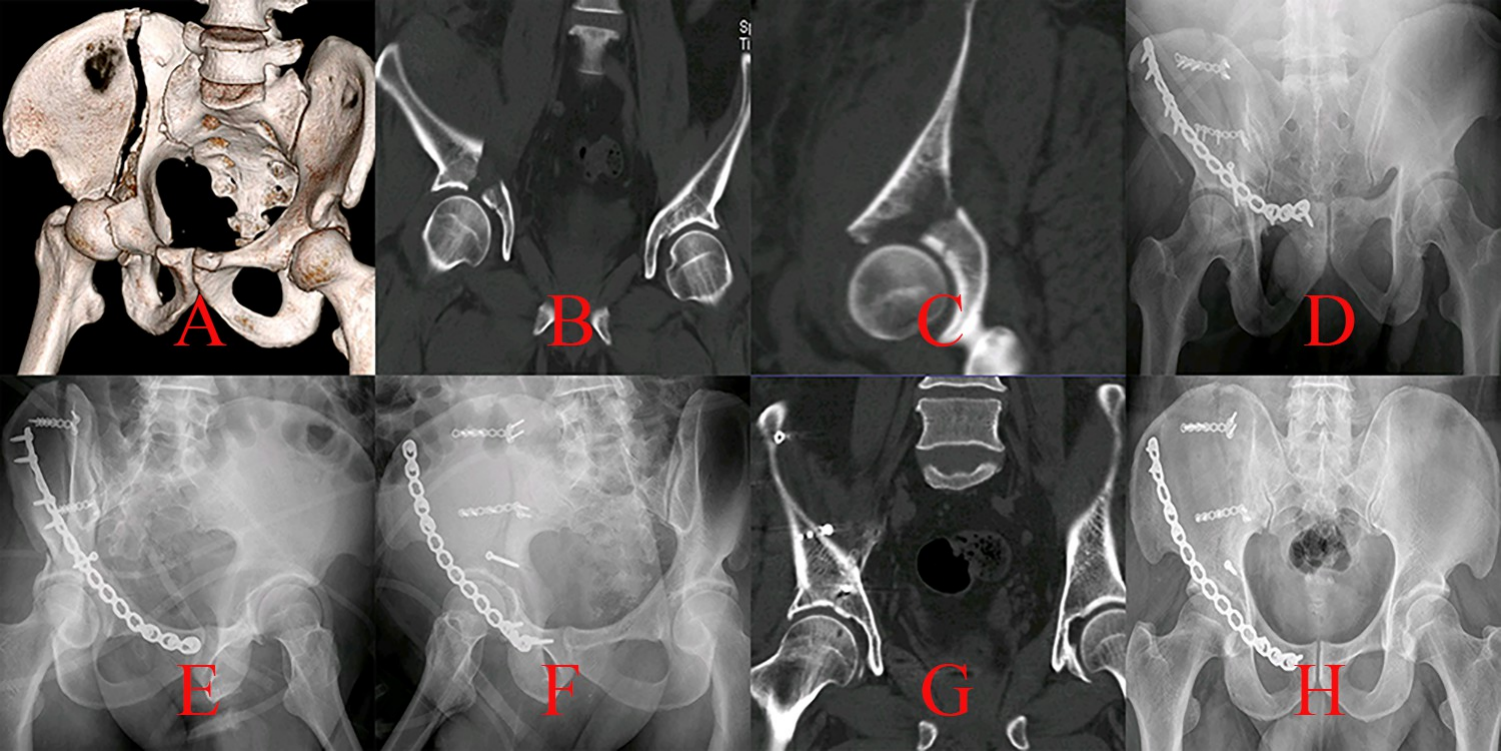
A case with A3.2 injury managed through the ilioinguinal technique was presented. A, cephalad and separated detachment of roof column on 3D reconstruction; B-C, cephalad and separated detachment lead to mismatch of acetabulum and femoral head on preoperative CT scans; D-F, the reconstructed acetabulum presented a larger diameter than the corresponding femoral heads and the contralateral acetabulum on postoperative images (imperfect reduction quality), an additional lag screw was placed to enhance the stability of roof articular surface; G, postoperative CT scan; H, AP view 3 months postoperatively.

**Fig 4:**
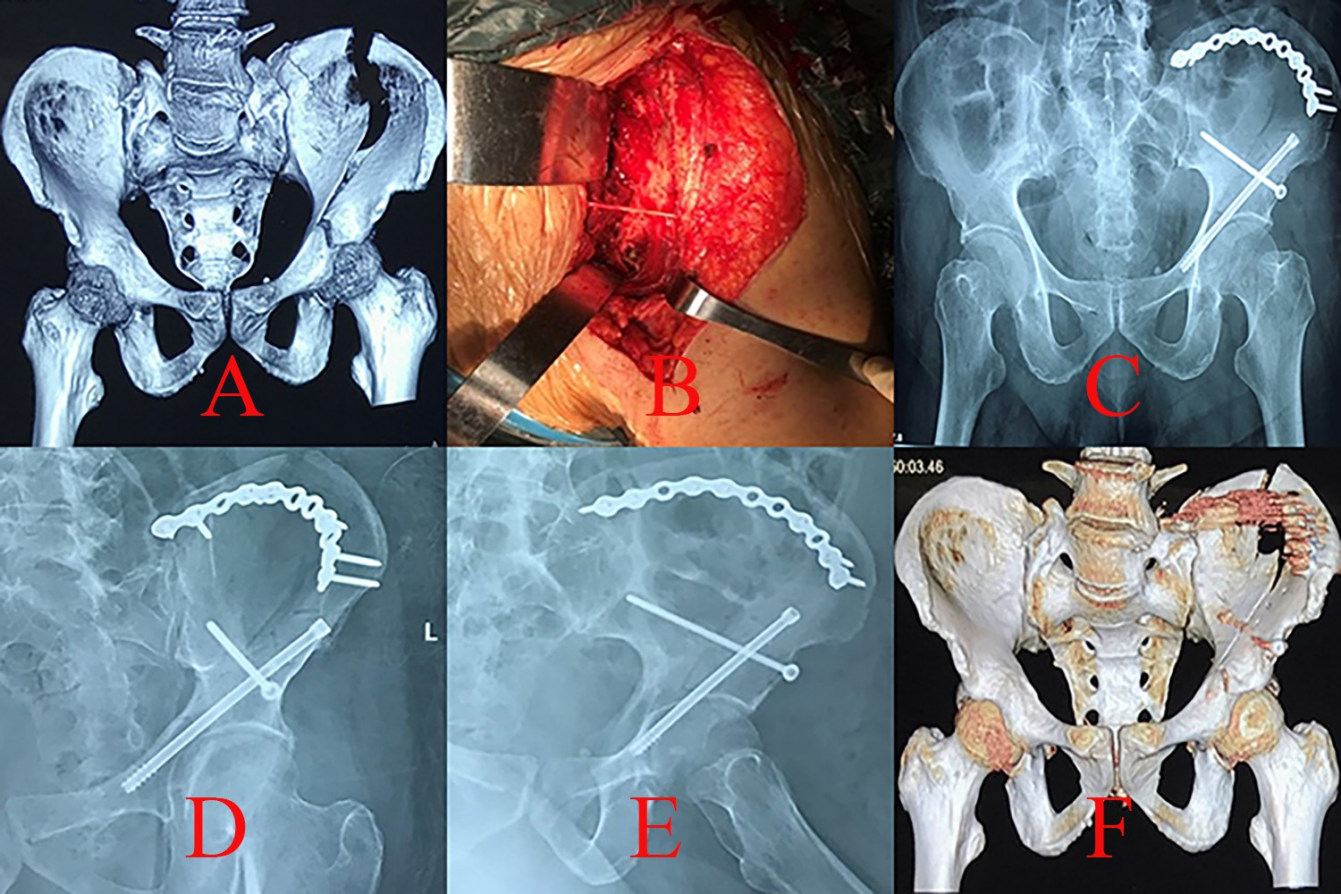
A case with A3.2 injury fixed with plating and lag screws through the iliofemoral approach was presented. A, cephalad and separated detachment of roof column on 3D reconstruction; B, surgical exposure of iliofemoral technique; C-E excellent concentric reduction on postoperative views; F, excellent reduction quality on postoperative 3D reconstruction.

**Fig 5:**
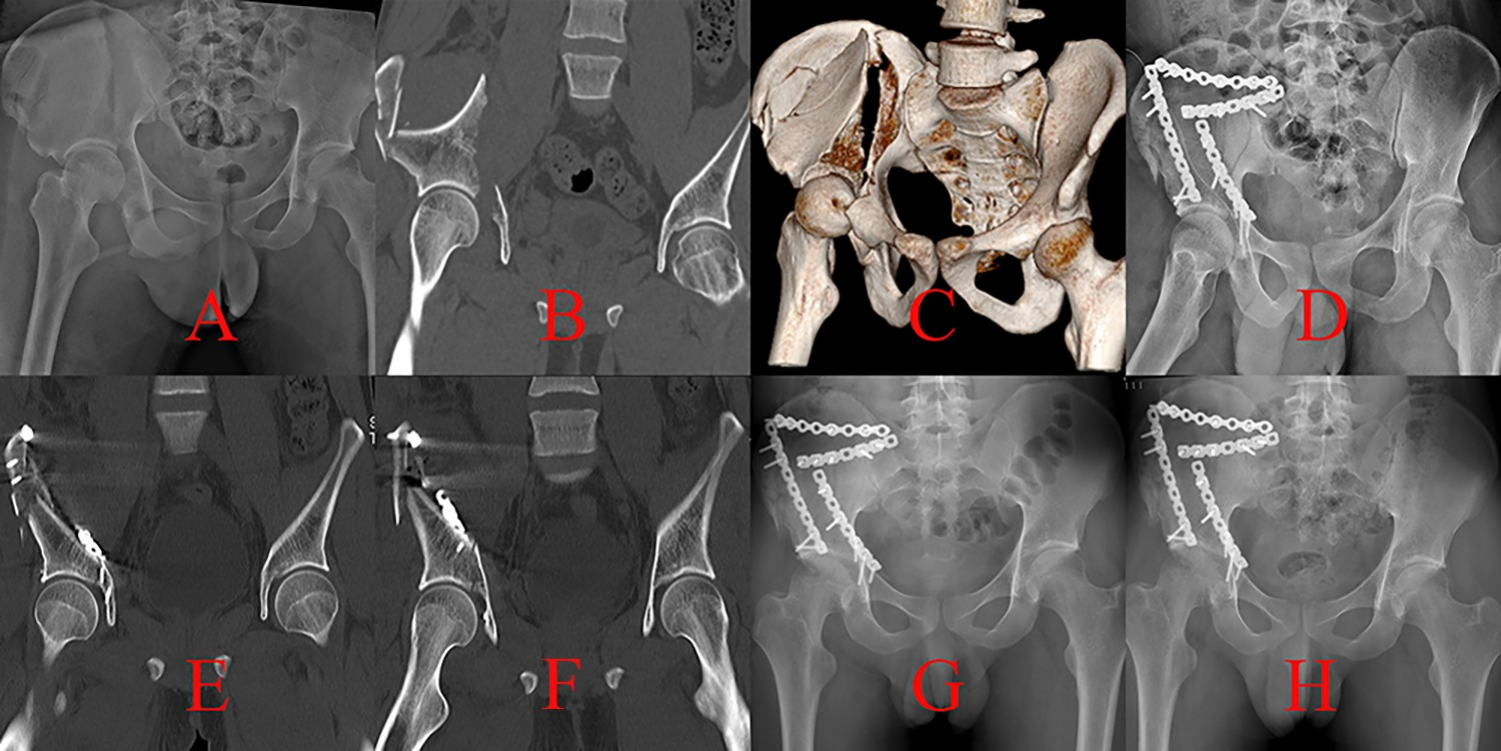
A patient with an A3.2 injury fixed with multi-plating through the iliac fossa technique was presented. A, cephalad detachment of roof column and intact iliopectineal line on preoperative AP view; B, mismatch of acetabulum (a larger diameter) and femoral head; C, cephalad and separated detachment of roof column on 3D reconstruction; D, excellent reduction quality on postoperative projection; E-F, postoperative CT scans showed excellent concentric reduction of acetabulum and femoral head; G, AP view a month postoperatively; H, fracture lines almost disappeared on AP view 3 months postoperatively.

Compared with A3.1 injury, the hip joint was more abducent during the impaction procedure for roof column detachment (A3.2). The fracture line of type A3.2 injury extended to the iliac crest from the articular roof surface; however, the pelvic brim was not involved [4]. Then, discontinuity of the iliopubic line could not be detected in preoperative radiographic examination [4, 5]. A relatively large fragment (cephalad and externally rotated displaced) attached to the acetabular roof could usually be observed in this fracture type, indicating a significant mismatch of the acetabulum and femoral head. Thus, it was a precondition for a good prognosis that an open reduction restores the height and corrects the rotation deformity of the displaced roof column. Roof column fracture was defined as a special form of posterior wall fracture, and KL technique was recommended in traditional Letournel-Judet classification system [1]. However, fracture lines involving the iliac wing in an A3.2 injury could not be adequately exposed through the KL incision [16]. Thus, poor reduction quality and unsatisfactory outcome may occur based on the Letournel-Judet classification and its treatment strategy. Iliac crest and entire iliac wing could be exposed through ilioinguinal, iliofemoral, and iliac fossa approaches. All approaches described above have been employed to manage roof column fractures in this study (Figs 3–5). Compared with other procedures, iliac fossa approach was a less invasive technique [15, 17, 18]. Thus, the iliac fossa procedure was preferred to manage A3.2 injury (Table 5). A relevant long fracture line or large fragment can usually be observed for some patients with roof column fractures, and a single reconstruction plate or lag screw may not provide sufficient stability for detached fragments [4, 5]. Therefore, multi-plating or plating with lag screw was essential to achieve firm fixation.

**Table 5:** Surgical strategies recommended based on the clinical results.

Fracture type	Surgical approach	Fixation method
A3.1	Iliofemoral	Plating and lag screw
A3.2	Iliac fossa	Multi-plating (or plating with lag screw)
A3.3	Combined/ Iliofemoral	Multi-plating

Surgical strategies recommended in this study are presented. Combined, iliac fossa and KL approach.

**Fig 6:**
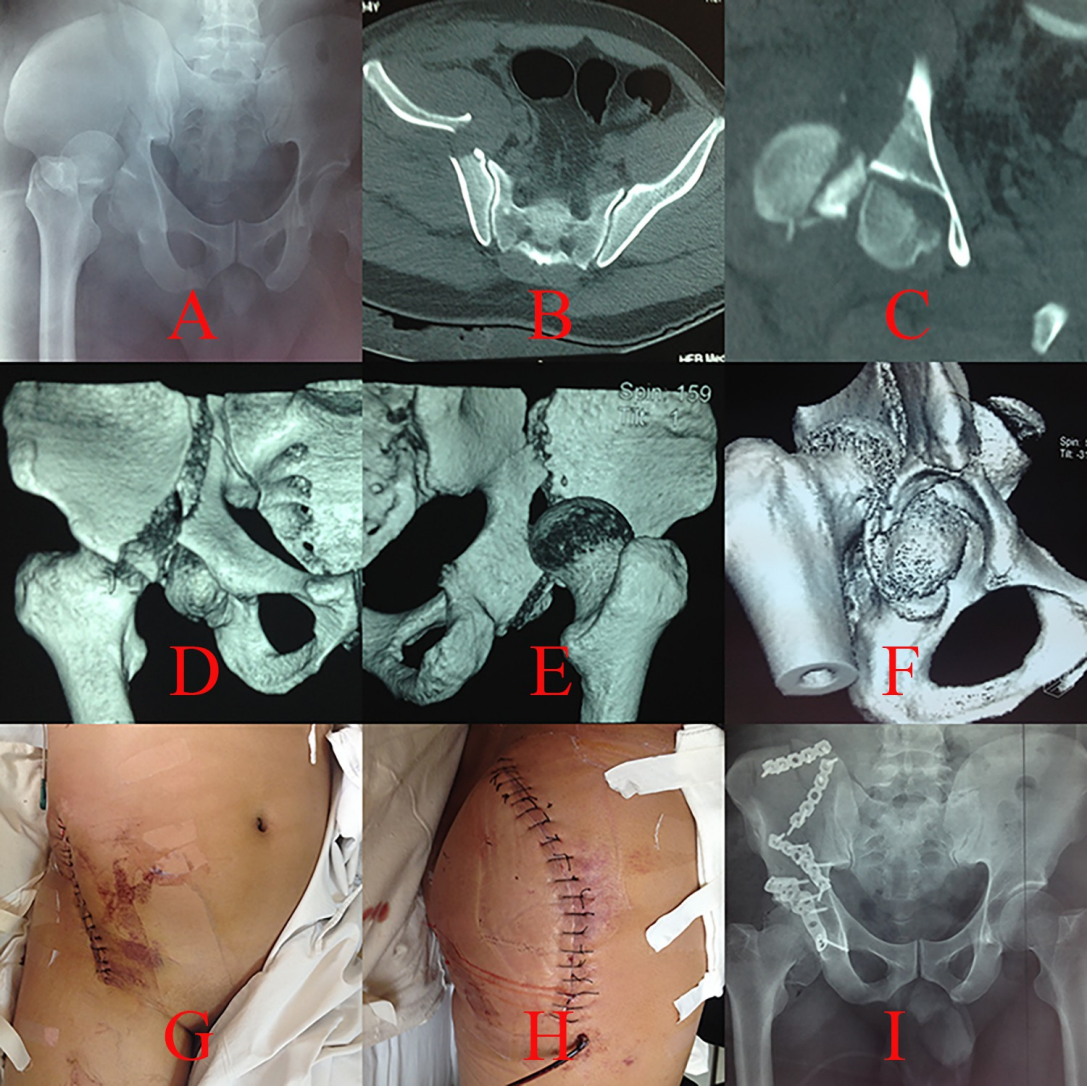
A patient with A3.3 injury managed through combined approaches was presented. A, cephalad detachment of roof column and femoral head with an intact iliopectineal line; B-C, detachment of roof column/wall and femoral head on CT scans; D-F, cephalad detachment of roof column/wall and femoral head dislocation on 3D reconstruction; G-H, surgical incisions (iliac fossa and KL); I, imperfect reduction quality on postoperative AP view.

Fracture lines may further involve the roof wall if the impaction energy was not completely released after roof column fracture (A3.2); then, complicated roof column fractures (A3.3) would occur. This fracture type was always characterized by both roof column and wall fractures, whose treatment was more challenging [4]. Adequate exposure and satisfactory reduction for complicated roof column fractures were difficult to accomplish through a single KL or iliac fossa approach; thus, combined incisions may be necessary (Fig 6). The majority of fracture lines in A3.3 injury could be managed through the iliofemoral technique, which was an alternative surgical procedure. Based on Three-column classification theory, roof column was directly connected with buttress column, and roof wall was raised from roof column [4]. Therefore, the management of fragments in roof columns should be superior to that in roof walls. The buttress column remains intact in complicated roof column fractures and serves as a reduction landmark for detached roof column fragments. Then, reduction of the roof wall fragment should be performed regarding the reduced roof column as an anatomical landmark.

**Fig 7:**
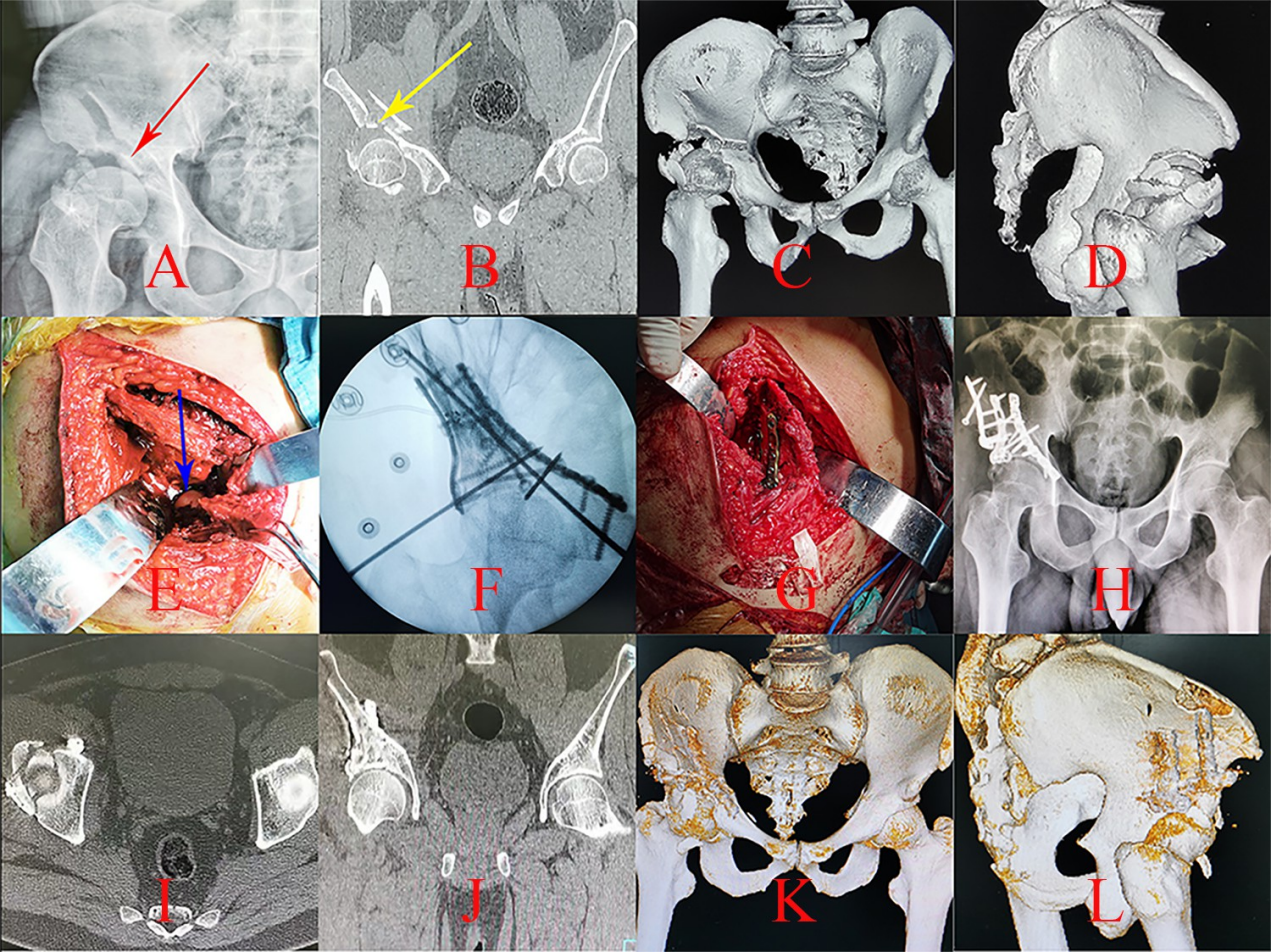
A 3.3 injury patient with articular surface compression who received iliac crest osteotomy through the iliofemoral technique was presented. A, gull sign (red arrow) in preoperative AP view; B, articular surface compression (yellow arrow) in preoperative CT scan; C-D, detachment of roof column and wall on preoperative 3D reconstruction; E, femoral head (blue arrow) could be observed after iliac crest osteotomy, which could serve as a landmark for the reduction of gull fragment; F, reduction of articular surface compression during intraoperative projection; G, intraoperative view of fixation; H, imperfect reduction quality on postoperative AP view; I-J, the reconstructed acetabulum presented a larger diameter than the corresponding femoral heads and the contralateral acetabulum on postoperative CT scans; K-L, fracture fixation on postoperative 3D reconstruction.

Articular surface compression may occur when the acetabular roof receives high energy impaction, especially for patients with osteoporosis [19, 20]. The gull sign (related to marginal impaction), indicating a mismatch of the acetabular roof and femoral head, could be observed during the preoperative radiographic examination [19]. Impaction of gull sign may be distinct because of different violence mechanisms. Anglen reported that gull sign involved superomedial impaction because the violence of femoral head on acetabulum was from inferolateral to superomedial [21]. However, the impaction of gull sign in this study was lateral. The reason for the difference was that the affected thigh was more adductive and the lateral roof was directly impacted during the fracture procedure in A3 injury. Surgical approaches to manage fragments would be also different based on the impaction location of gull sign. Osteotomy of the iliac crest through iliofemoral incision was an optional technique to adequately expose the compressed articular surface (Fig 7). Regarding the femoral head as a landmark, detached articular roof surface fragments should be anatomically reduced through the iliofemoral approach [19]. For patients with a large fragment, lag screw or plating was recommended to stabilize the “gull” fragment. However, screw insertion may be inhibited if the “gull” fragment was excessively small and thin (yellow arrow in Fig 7B). Friction fit and adequate subarticular buttress were needed to maintain the stability of “gull” fragment. Bone graft under articular surface should be performed for patients with bone defects [20]. Screw penetration into hip joint during fragment fixation must be avoided to avoid causing postoperative traumatic arthritis. Lin Z et al conducted a fluoroscopic study and concluded that acetabular lateral projection was an ideal fluoroscopic method to assess the accuracy of screw placement [22].

The majority of cases (76.67%) in this study obtained satisfactory functional outcomes because the surgical strategies of all patients were developed by experienced surgeons based on Three-column classification. Our experience and lessons regarding the treatment of acetabular roof fractures were presented for orthopedic surgeons (Table 5). However, there were some limitations in this study. The incidence of acetabular roof fractures was relatively low, only 60 patients were included at 9 Level-1 trauma centers from January 2015 to 2019 January. As a retrospective study, patients were not randomly recruited, the completeness and accuracy of the data may be affected, posing a risk of data bias, then, the limited sample size may not represent the relevant traits of all roof column/wall fractures. Additionally, a comparison of different surgical procedures was not conducted because of limited sample size in this investigation, which limits the exploration of the best surgical strategy. All patients completed at least 2 years of follow-up in this study, however, detailed assessment of long-term prognosis and complications may be insufficient. In the future, we will include more patients for prospective comparative studies with long follow-up time to further explore the treatment strategy of roof injury.

## Conclusion

The injury mechanism of A3 fractures in Three-column classification was the direct impaction from femoral head on acetabular roof. Reduction and fixation of A3 injury were technique demanding, and poor prognosis may be accompanied even treated by experienced surgeons. Appropriate surgical strategies (Table 5) based on fracture characteristics in three subtypes of A3 injury were the premise of accepted prognosis.

## Supporting information

S1 Data Relevant original data used in this research was presented in S1 Data. (XLSX)
